# Hydroxychloroquine and a low activity bisphosphonate conjugate prevent and reverse ovariectomy-induced bone loss in mice through dual antiresorptive and anabolic effects

**DOI:** 10.21203/rs.3.rs-4237258/v1

**Published:** 2024-05-03

**Authors:** Zhenqiang Yao, Akram Ayoub, Venkatesan Srinivasan, Jun Wu, Churou Tang, Rong Duan, Aleksa Milosavljevic, Frank Ebetino, Alison Frontier, Brendan Boyce

**Affiliations:** University of Rochester Medical Center; University of Rochester Medical Center; Department of Chemistry, University of Rochester; University of Rochester Medical Center; University of Rochester Medical Center; university of Rochester; Department of Chemistry, University of Rochester; Department of Chemistry, University of Rochester; Department of Chemistry, University of Rochester; University of Rochester Medical Center

**Keywords:** osteoporosis, osteoclast, osteoblast, hydroxychloroquine, bisphosphonate, TRAF3

## Abstract

Osteoporosis is incurable because there are no dual antiresorptive and anabolic therapeutic agents that can be administered long-term. The most widely used antiresorptive agents, bisphosphonates (BPs), also inhibit bone formation and thus have limited effect in preventing osteoporotic fracture. Hydroxychloroquine (HCQ), which is used to treat rheumatoid arthritis, prevents the lysosomal degradation of TNF receptor-associated factor 3 (TRAF3), an NF-κB adaptor protein that limits bone resorption and maintains bone formation. We attempted to covalently link HCQ to a hydroxyalklyl BP (HABP) with anticipated low antiresorptive activity, to target delivery of HCQ to bone to test if this targeting increases its efficacy to prevent TRAF3 degradation in the bone microenvironment and thus reduce bone resorption and increase bone formation, while reducing its systemic side effects. Unexpectedly, HABP-HCQ was found to exist as a salt in aqueous solution, composed of a protonated HCQ cation and a deprotonated HABP anion. Nevertheless, it inhibited osteoclastogenesis, stimulated osteoblast differentiation, and increased TRAF3 protein levels in vitro. HABP-HCQ significantly inhibited both osteoclast formation and bone marrow fibrosis in mice given multiple daily PTH injections. In contrast, HCQ inhibited fibrosis, but not osteoclast formation, while the HABP alone inhibited osteoclast formation, but not fibrosis, in the mice. HABP-HCQ, but not HCQ, prevented trabecular bone loss following ovariectomy in mice and, importantly, increased bone volume in ovariectomized mice with established bone loss because HABP-HCQ increased bone formation and decreased bone resorption parameters simultaneously. In contrast, HCQ increased bone formation, but did not decrease bone resorption parameters, while HABP also restored the bone lost in ovariectomized mice, but it inhibited parameters of both bone resorption and formation. Our findings suggest that the combination of HABP and HCQ could have dual antiresorptive and anabolic effects to prevent and treat osteoporosis.

## Introduction

Osteoporosis is a major disease of aging characterized by decreased bone mass and strength, resulting in increased fracture risk. About 44 million people in the U.S. have low bone mineral density (BMD), and 10 millions of them are osteoporotic ^1^. Elderly patients with femoral neck fractures develop serious complications, including pneumonia and deep vein thrombosis due to prolonged bed rest, and up to 20 % of them die within the 12 months after the fracture ^2^.

Osteoporosis is caused by imbalanced bone remodeling, due to enhanced bone resorption and relatively reduced bone formation, mediated by osteoclasts (OCs) and osteoblasts (OBs) respectively ^1^. Both antiresorptive and anabolic agents are available, but they do not satisfactorily treat osteoporosis. For example, the antiresorptive agents, bisphosphonates (BPs) and the RANKL monoclonal antibody, denosumab, also inhibit bone formation ^3,4^, but they reduce the rate of osteoporotic fracture by only ~50% ^5^ and a small percentage of patients also develop osteonecrosis of the jaw or atypical femoral shaft fractures^6^. In addition, following discontinuation of denosumab some patients have vertebral fractures due to rebound increased bone resorption ^7,8^. Furthermore, treatment with anabolic agents is limited to two years due to safety concerns ^9–12^, and is typically followed by treatment with antiresorptive agents ^13^, because their anabolic effects are transient, and discontinuation results in increased bone resorption ^14^. In addition, combination therapy with teriparatide and a BP does not appear to offer advantages over the use of either agent alone ^15,16^. Similarly, switching from alendronate to teriparatide does not improve hip BMD, although addition of an anabolic agent to ongoing alendronate treatment does ^17^. The recently approved sclerostin monoclonal Ab, romosozumab, transiently increases bone formation and inhibits bone resorption in osteoporotic patients ^18^. However, treatment is associated with severe side effects, including myocardial infarction, stroke and cardiovascular death ^11^, and is recommended for up to only 1 year in patients with severe osteoporosis ^19^. Therefore, there is an unmet need to develop a new strategy to prevent and treat osteoporosis.

BPs are characterized by two phosphate groups joined to a central carbon atom forming a stable, degradation-resistant phosphomethylene moiety with two potential side chains (R1 and R2). For most of the clinically used BPs, including zoledronate, alendronate, ibandronate, risedronate, and pamidronate, one side chain is -OH, which adds binding affinity to bone mineral, and another side chain introduces a basic nitrogen moiety via an alkyl chain or a heterocyclic group to enhance their antiresorptive activity ^20^. Consequently, these are called the nitrogen-containing BP (N-BP) class of drugs. It is now clear that the N-BPs inhibit farnesyl pyrophosphate synthase (FPPS) ^21,22^, in particular in osteoclasts (OCs), resulting in disruption of cytoskeletal organization, loss of OC ruffled border formation, altered vesicular trafficking, and apoptosis ^23^. The distance and orientation of the nitrogen moiety relative to the phosphonate groups (for example, the length of the aminoalkyl chain or the position of the nitrogen atom within a heterocyclic ring) markedly influence the antiresorptive potency of N-BPs^24^. By virtue of calcium-chelating properties, the phosphonate groups of BPs can deposit tightly in bone, and thus a druggable agent that is linked to a BP can be targeted for delivery to bone specifically to treat bone diseases more efficiently, while reducing systemic side effects of the drugs ^25,26^. A group of BP analogs, for example, hydroxyalklyl BP (HABP), have been developed for the specific use for targeting druggable agents to bone ^25,26^. Our aim was to develop a BP with low resorptive activity conjugated to therapeutic agents with potential dual antiresorptive and anabolic effects, such as hydroxychloroquine (HCQ), and target them to bone to treat osteoporosis.

HCQ, an anti-inflammatory drug that is used to treat malaria and rheumatoid arthritis, could potentially be a dual antiresorptive and anabolic agent by preventing the lysosomal degradation of TNF receptor-associated factor 3 (TRAF3), an adaptor protein that transduces intracellular signaling of cytokines, including RANKL^27,28^, TNFa^27,28^, CD40L^29^ and TGFb ^30^. TRAF3 negatively regulates OC formation by limiting non-canonical processing of NF-kB p100 to p52 in OC precursors ^27,28^, and maintains osteoblast (OB) differentiation by limiting TGFb-induced GSK3b-mediated b-catenin degradation in OB precursors ^30^. Conditional knockout (cKO) of TRAF3 in either myeloid or OB lineage cells resulted in early onset osteoporosis in mice due to increased bone resorption and reduced bone formation, respectively ^30,31^. However, HCQ can have systemic side effects, such as cardiac toxicity and blindness, in up to 0.5–1% of patients ^32,33^. We therefore sought to link HCQ to a BP analog with low resorptive activity with the goal to administer an overall lower dose of HCQ as a long-term treatment of osteoporosis, while delivering a relatively high concentration to bone and thus reducing its systemic side effects because the BP would target HCQ to bone where it would be released in the acidic microenvironment of resorption lacunae at a locally higher concentration. HCQ was covalently linked to a BP analog, HABP, as in the synthesis of a bone-targeted chloroquine conjugate ^25^. Unexpectedly, the compound was found to exist as a salt in water, composed of deprotonated HABP anions and protonated HCQ cations (Fig. S1 to S5). However, it is interesting that this combination of HCQ and HABP effectively prevented bone loss and restored the bone lost in ovariectomized mice due to antiresorptive and anabolic effects mediated by HABP and HCQ, respectively. HCQ alone, even at high dose, did not prevent or treat ovariectomy-induced bone loss, although it stimulated bone formation. The HABP alone restored the bone lost in ovariectomized mice by inhibiting bone resorption, but like other BPs, it also inhibited bone formation. Thus, a combination of low dose HCQ and HABP could be effective as a long-term treatment of osteoporosis by inhibiting bone resorption and stimulating bone formation simultaneously.

## Results

### HABP-HCQ inhibits OC formation and stimulates OB differentiation *in vitro*.

**Figure 1:**
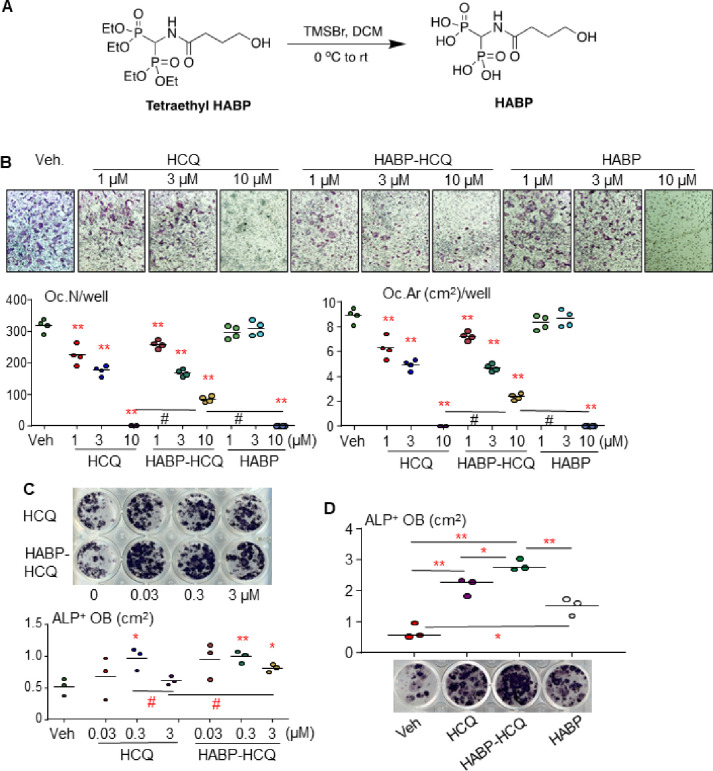
HABP-HCQ stimulates OB and inhibits OC differentiation. (A) Synthesis of HABP – Hydrolysis of tetraethyl HABP with bromotrimethylsilane. (B) C57Bl6 mouse BM cells were cultured with M-CSF for 2 d followed by treatment with RANKL plus the indicated doses of HCQ, HABP-HCQ, or HABP for 3 d. OC number and surface were evaluated after TRAP staining. (C) BM cells were cultured for 5 d in 24-well plates to expand stromal cells, which were induced for OB differentiation in the presence of the indicated doses of HCQ or HABP-HCQ intermittently, 6 h each d, for 5 d. ALP staining was performed to evaluate OB differentiation. (D) Effects of 1 mM HABP vs. 1 mM HABP-HCQ and HCQ on OB differentiation, tested in 12-well plates, as above in (B). *p<0.05 & **p<0.01 vs. vehicle, or #p<0.01; one-way ANOVA+Dunnett’s test.

Bone marrow (BM) cells were cultured with M-CSF for 2 d to increase OC precursor numbers. 10 ng/ml RANKL and different doses of HCQ, HABP obtained from the hydrolysis of the phosphonate ester tetraethyl HABP (Fig. 1A), or combinations of them were then added to the cultures for 3 d. TRAP staining was performed to evaluate OC formation. Like HCQ, HABP-HCQ inhibited RANKL-induced OC formation from 1 mM and completely suppressed OC formation at 10 mM (Fig. 1B). When the cultures were stopped early (RANKL treatment for 2.5 d), we found that OC numbers and areas in the wells treated with 10 mM HABP-HCQ were higher than those treated with the same dose of HCQ (Fig. 1B). HABP alone did not inhibit OC formation at 1 or 3 mM, but completely inhibited OC formation at 10 mM (Fig. 1B).

BM cells from C57Bl6 mice were cultured for 5 d to expand stromal cells. The cells were then induced for OB differentiation in the presence of HCQ or HABP-HCQ. Interestingly, the concentration of HCQ and HABP-HCQ that stimulated OB differentiation was lower than that required to inhibit OC formation, starting around 0.03 mM (Fig. 1C). However, higher concentrations of HCQ (3 mM) tended to inhibit OB differentiation (Fig. 1C), and this inhibition was attenuated by the combination of HCQ and HABP (Fig. 1C) because the HABP also slightly promoted OB differentiation from the BM stromal cells (Fig. 1D).

**Figure 2:**
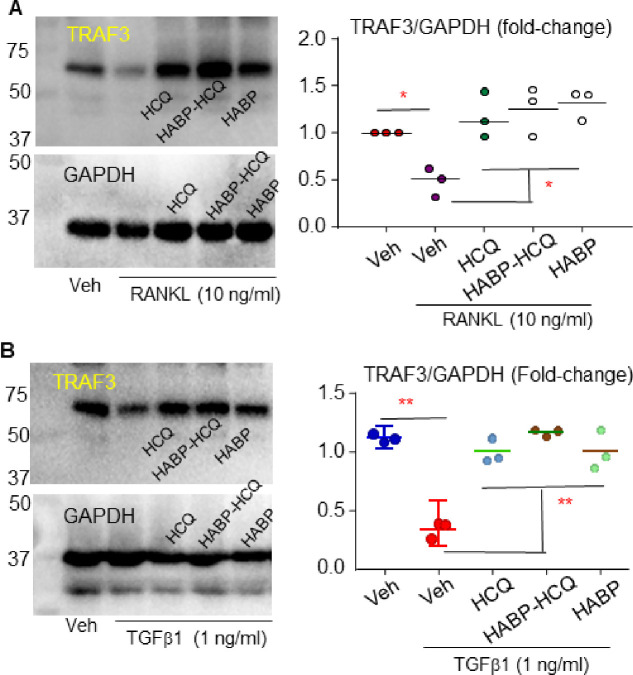
HABP-HCQ stabilizes TRAF3 in OC and OB progenitor cells. (A) C57Bl6 mouse BM cells were cultured with M-CSF for 3 d to expand macrophages, which were treated with vehicle or RANKL plus 3 uM of the indicated compounds for 24 hr. Protein levels of TRAF3 and its related level normalized to actin were analyzed by WB. (B) BdMPCs were treated with TGFb1 plus 3 uM of the indicated compounds for 24 hr, and levels of TRAF3 and actin protein were analyzed by WB. *p<0.05 & **p<0.01 vs. vehicle, #p<0.01 between the two groups, one-way ANOVA+/Dunnett’s test.

RANKL and TGFb1 induce TRAF3 ubiquitination (Ub) and lysosomal degradation in myeloid cells and MPCs to promote OC and inhibit OB differentiation, respectively ^28,30,31^. Like HCQ, the HABP-HCQ prevented RANKL-induced TRAF3 degradation in OC precursors (Fig. 2A). Similarly, both HCQ and HABP-HCQ prevented TGFb1-induced TRAF3 degradation in MPCs (Fig. 2B). Interestingly, the HABP also prevented RANKL-induced TRAF3 degradation in OC precursors (Fig. 2A).

### HABP-HCQ prevents PTH-induced OC formation and BM fibrosis in vivo in mice.

**Figure 3:**
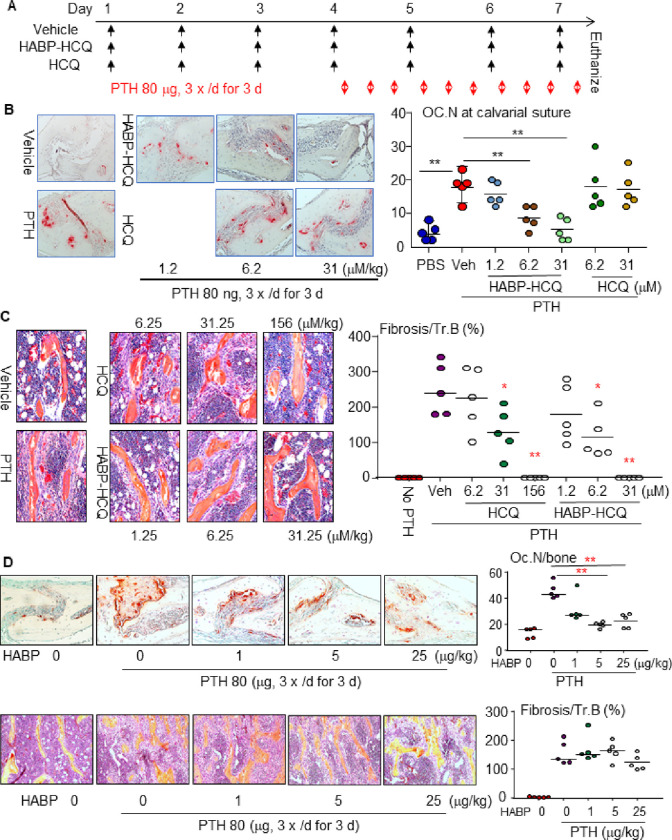
Low dose of HABP-HCQ effectively prevents PTH-induced OC formation and BM fibrosis in mice. (A) Diagram showing the treatment schedule of HABP-HCQ vs HCQ and PTH in mice. (B) TRAP-stained calvarial bones were used to quantify OC number (upper panel) and H&E-stained tibial bones were used to quantify fibrosis around trabeculae (lower panel). (C) An independent experiment was performed to evaluate the effect of HABP on PTH-induced OC formation in calvarial bones (upper panel) and BM fibrosis in tibial bones (lower panel). 5 mice per group for all experiments. *p<0.05, **p<0.01 vs. Vehicle or the two groups with line, one-way ANOVA+Dunnett’s test.

Intermittent single daily injections of PTH stimulate bone formation, while multiple daily injections of PTH, like hyperparathyroidism, cause significant bone erosion due to enhanced OC formation ^34^. We evaluated the dose of HABP-HCQ required to inhibit OC formation induced by multiple daily injections of PTH into the subcutaneous tissues overlying the calvarial bones of C57B6 mice ^31^ (Fig. 3A). As expected, multiple daily PTH injections significantly increased OC numbers in the calvarial BM and suture area (Fig. 3B). Neither 6 nor 30 mM/kg HCQ inhibited PTH-induced OC formation in calvarial bones (Fig. 3B), suggesting that >30 mM/kg HCQ is required to inhibit OC formation. In contrast, HABP-HCQ significantly inhibited PTH-induced OC formation in calvarial bones at 6 and 30 mM/kg, but not at 1.25 mM/kg (Fig. 3B), suggesting that ~6 mM/kg HABP-HCQ is required to effectively inhibit PTH-induced OC formation in mice.

Multiple daily injections of PTH also cause BM fibrosis ^31^. HCQ (31.25 and 156, but not 6.25 mM/kg) blocked PTH-induced BM fibrosis (Fig. 3C). In contrast, HABP-HCQ (from 6.25 mM/kg) blocked PTH-induced BM fibrosis (Fig. 3C). Interestingly, the multilayered proliferation of fibroblastic/spindle cells on tibial trabecular surfaces in mice treated with multiple daily doses of PTH was prevented by 156 mM/kg HCQ and 31.25 mM/kg HABP-HCQ, and the mice had only a single layer of osteoblastic cells on trabecular surfaces (Fig. 3C). We also evaluated the effect of the HABP on PTH-induced OC formation and BM fibrosis and found that it inhibited PTH-induced OC formation in the calvarial bones, starting ~5 mg/kg, a dose comparable to that of HABP-HCQ to inhibit OC formation (Fig. 3B upper panel). However, the HABP did not inhibit PTH-induced BM fibrosis (Fig. 3D lower panel).

### HABP-HCQ prevents bone loss in ovariectomized mice by inhibiting bone resorption and maintaining bone formation, partially dependent on TRAF3.

**Figure 4:**
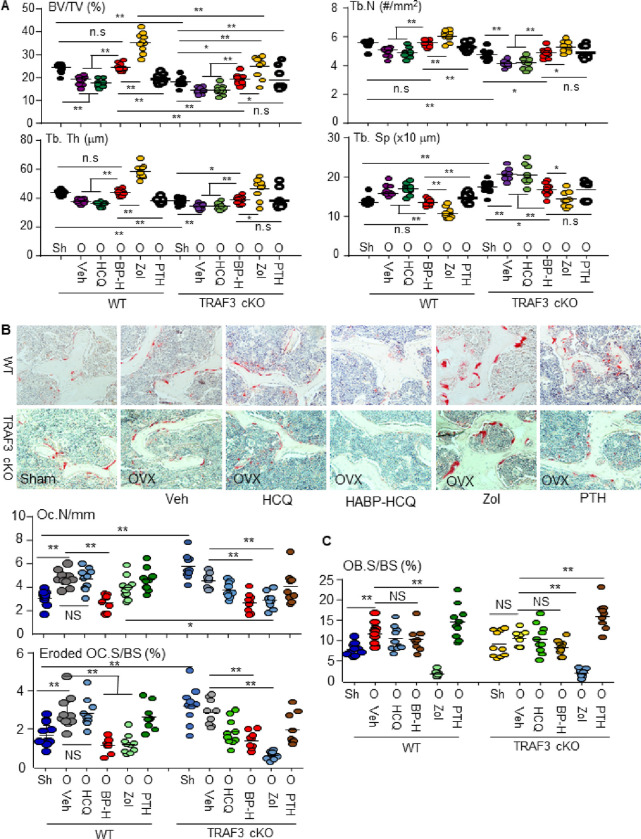
HABP-HCQ prevents OVX-induced bone loss independent of TRAF3. 10-wk-old female TRAF3^f/f^ and TRAF3^f/f^ LysM^cre^ (cKO) mice on a C57Bl6 background had sham or OVX surgery. From the 3^rd^ d, OVX mice for each phenotype were randomly assigned to 5 treatment groups, which were given 5 doses per wk of vehicle, HCQ (31.25 mM/kg), HABP-HCQ (6.25 mM/kg) or PTH (80 ng/mouse) or 1 weekly dose of zoledronate acid (0.15 mg/kg) for 5 wk. (A) The spines were micro-CT scanned to quantify the bone structural parameters in L1 vertebrae. BP-H = HABP-HCQ, Zol = zoledronate acid. (B) TRAP-stained L4 vertebrae was used to quantify OC number and surface on eroded surfaces. (C) OB surfaces on trabeculae were quantified on H&E-stained sections from L4 vertebrae. 8–9 mice per group for all experiments. *p<0.05, **p<0.01, one-way ANOVA+Dunnett’s test.

The dose of HCQ (£5 mg/kg) prescribed for long-term treatment of rheumatoid arthritis and other autoimmune diseases in humans ^35^ corresponds to 26.4 mg (60 mM)/kg in mice ^36^. We administrated 6.25 mM/kg HABP-HCQ (containing 2.6 mg/kg HCQ) or 31.25 mM/kg (containing 13.6 mg/kg) HCQ as starting doses to test if the lower dose of HABP-HCQ could prevent OVX-induced bone loss and if this depends on TRAF3 expression in WT (TRAF3^f/f^) and myeloid cell TRAF3 conditional knockout (TRAF3^f/f^LyM^cre^, TRAF3 cKO) mice ^31^. As expected, OVX significantly decreased trabecular bone mass in the vertebrae of WT (TRAF3^f/f^) mice due to reduced Tb.N and Tb.Th compared to sham WT mice (Fig. 4A). Consistent with the general concept that postmenopausal osteoporosis is characterized by high bone turnover, OVX resulted in increased OC numbers and surfaces as well as increased OB surfaces in vertebral bone sections from WT mice (Fig. 4B&C).

5 doses/week of 6.25 mM/kg HABP-HCQ significantly increased vertebral bone mass in ovariectomized WT mice to values that were similar to those in sham mice (Fig. 4A). Interestingly, HABP-HCQ not only significantly reduced OC numbers and surfaces, but also maintained OB surfaces at a high level in vertebral sections from ovariectomized mice (Fig. 4B & C). However, a 5-fold higher dose (31.25 mM) of HCQ did not increase vertebral BV/TV in the ovariectomized WT mice (Fig. 4A) probably because it did not reduce OC numbers or surfaces (Fig. 4B), although it maintained increased OB surfaces in the ovariectomized mice (Fig. 4C).

As a positive antiresorptive control, weekly injections of 150 mg/kg zoledronic acid significantly increased vertebral BV/TV, Tb.N and Tb.Th (Fig. 4A) in ovariectomized WT mice. Of note, some large osteoclasts were present on the trabecular surfaces and in the BM space in zoledronic acid-treated mice (Fig. 4B), which have been reported in animals ^37^, and are similar to the non-functional, “giant osteoclasts” described in bone samples from humans treated with bisphosphonates ^38^. We concluded that these giant osteoclasts in zoledronic acid-treated mice were non-functional because bone surfaces with active resorption lacunae were reduced (Fig. 4B). This is consistent with the report that bisphosphonates blocks prenylation of small GTPases ^39^, causing disruption of cytoskeletal organization, vesicle transport, loss of the sealing zone, and ruffled border ^40,41^, resulting in the detachment of osteoclasts from bone surfaces ^37^. Zoledronic acid also significantly reduced OB surfaces (Fig. 4C), as expected. As a positive anabolic control, PTH did not increase BV/TV in WT ovariectomized mice at this stage, probably because the OVX mice already had increased OB surfaces, and PTH did not further increase these (Fig. 4C) and did not change OC parameters either (Fig. 4B).

BV/TV values in the mice with TRAF3 cKO in myeloid cells were significantly lower than those in WT control mice (Fig. 4A), as expected. OVX further decreased BV/TV values (Fig. 4A), but it did not further increase OC numbers or eroded surfaces (Fig. 4B) in TRAF3 cKO mice likely because these resorptive parameters were already elevated. OVX did not change OB surfaces in TRAF3 cKO mice (Fig. 4C). As in the WT ovariectomized mice, HABP-HCQ, but not HCQ alone, significantly increased vertebral BV/TV values, associated with increased Tb. N and Tb. Th and reduced OC numbers and eroded surfaces in the ovariectomized TRAF3 cKO mice (Fig. 4B). However, vertebral BV/TV values in the ovariectomized TRAF3 cKO mice treated with HABP-HCQ were still markedly lower than that in WT mice. In addition, neither HCQ nor HABP-HCQ changed OB surfaces in the ovariectomized TRAF3 cKO mice (Fig. 4C). These findings suggest that HABP-HCQ-induced elevation of bone mass is partially dependent on the expression of TRAF3. This is consistent with the findings that HABP-HCQ prevents TRAF3 degradation induced by RANKL and TGFb1 in myeloid cells and MPCs, respectively (Fig. 2A & B). Weekly doses of 150 mg/kg zoledronic acid did not reduce the number of giant OCs in the TRAF3 control floxed mice (Fig. 4B), but similar to its effect in the ovariectomized TRAF3 cKO mice, it reduced OB surfaces in the floxed control mice (Fig. 4C). Intermittent PTH significantly increased spinal BV/TV values in the ovariectomized TRAF3 cKO mice (Fig. 4A), associated with increased OB surfaces (Fig. 4C), but it did not change OC parameters (Fig. 4B) because the OVX floxed mice already had increased OC surfaces and PTH did not further increase them (Fig. 4C).

### HABP-HCQ reverses lost bone in ovariectomized mice by inhibiting bone resorption and increasing bone formation.

**Figure 5:**
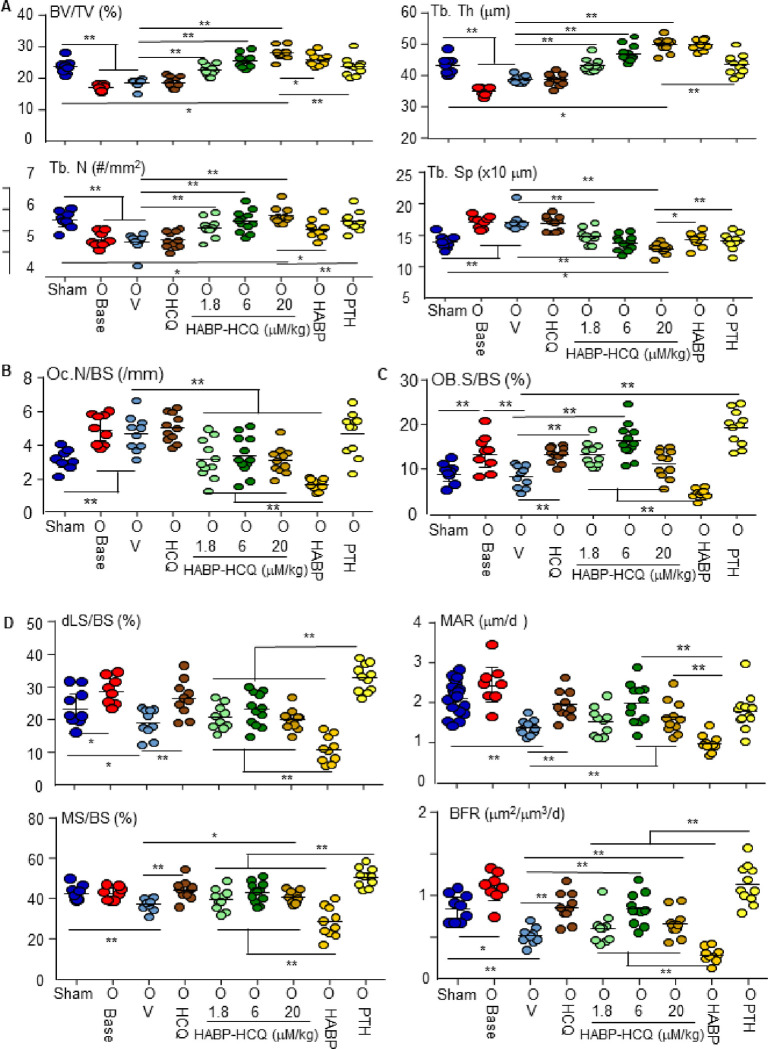
Low-dose HABP-HCQ restores the lost bone caused by OVX. 10-wk-old female C57Bl6 mice had sham (1 group) or OVX surgery (8 groups; 8–9 mice per group. 4 wk post-surgery, the sham and one group of ovariectomized mice were micro-CT scanned alive to confirm bone loss in ovariectomized mice (which then were euthanized). From the 5^th^ wk, the 8 groups of ovariectomized mice were randomly assigned to 5 doses per wk of the indicated treatments for 5 wk. (A) The spines were micro-CT scanned to quantify the bone structural parameters in L1 vertebrae. (B) TRAP-stained sections from L4 vertebrae were used to quantify OC numbers and surfaces on eroded surfaces. (C) OB surfaces on trabeculae were quantified on H&E-stained sections of L4 vertebrae. (D) Dynamic bone formation parameters were measured in plastic-embedded undecalcified sections from L1 vertebrae. *p<0.05, **p<0.01, one-way ANOVA+Dunnett’s test.

We tested the effect of the HABP-HCQ on established bone loss. 5 wk post OVX, bone loss was established in mice, as confirmed by micro-CT analysis (Fig. 5A). We then treated the mice with HCQ, three doses of HABP-HCQ, HABP, or intermittent PTH for 5 wk. HCQ, even given at high dose (20 mM/kg), did not increase vertebral bone mass. HABP-HCQ dose-dependently restored the lost bone in ovariectomized mice to or above the level in sham mice (Fig. 5A). HABP (1.8 mM/kg) and intermittent PTH also restored the lost bone in the OVX mice to the level in sham mice (Fig. 5A).

OVX increased OC numbers (Fig. 5B) and surfaces (Fig. S6), but HCQ did not change these parameters (Fig. 5B). In contrast, all doses of HABP-HCQ markedly reduced OC numbers (Fig. 5B) and surfaces (Fig. S6) in the ovariectomized mice, but their effects were lower than those of HABP alone (Fig. 5B and S6). As expected, PTH did not reduce OC numbers (Fig. 5B) or surfaces (Fig. S6).

OB surfaces were increased 4 wk after OVX and returned to the level of sham mice following 5 wk of treatment (9 weels after OVX) with vehicle (Fig. 5C). Interestingly, HCQ and the lowest dose of HABP-HCQ significantly increased OB surfaces in ovariectomized mice, while the highest dose of HABP-HCQ did not change OB surfaces (Fig. 5C). In contrast, HABP markedly reduced OB surfaces (Fig. 5C). As a positive control, PTH significantly increased OB surfaces in the ovariectomized mice (Fig. 5B). Like HCQ, HABP-HCQ also markedly increased dynamic bone formation parameters, including double-labelled surfaces, mineralizing surfaces, MARs and BFRs in the ovariectomized mice (Fig. 5D). HABP alone decreased, while PTH increased these dynamic parameters of bone formation (Fig. 5D), as expected.

## Discussion

Here, we report a dual anabolic and anticatabolic effect of a combination of HABP and hydroxychloroquine (HCQ) that effectively prevented ovariectomy-induced vertebral trabecular bone loss and restored bone lost in ovariectomized mice. We generated the HABP-HCQ conjugate with the aim of targeting HCQ to bone by conjugating it to a HABP to deliver an overall higher dose of HCQ to the bone microenvironment than would be delivered by HCQ alone, and to reduce potential side effects of the dose of HCQ typically administered to patients to treat rheumatoid arthritis. However, we discovered unexpectedly that HCQ dissociated from the HABP when the conjugate was dissolved in water. Nevertheless, the combination of HABP and HCQ was more effective at preventing or restoring bone loss after ovariectomy than either alone. We attributed the antiresorptive effects of the HABP-HCQ primarily to the HABP, rather than HCQ, because: 1) the lowest effective dose in vitro of HCQ (3 mM) that inhibited OC formation is high and it would have been necessary to deliver very large doses of the drug to reach this threshold dose in vivo in mice; 2) HCQ did not reduce bone resorption parameters in ovariectomized mice or in the multiple PTH injection model; and 3) HABP inhibited bone resorption in ovariectomized mice and in the PTH multiple injection mouse model. We attribute the anabolic effects of HABP-HCQ to HCQ because: 1) the dose of HCQ to stimulate OB differentiation in vitro is as low as 0.03 mM and the combination of HCQ and HABP is better than HCQ alone to stimulate OB differentiation in vitro (Fig. 1C&D); 2) the combination of HCQ and HABP, like HCQ alone, increased bone formation parameters in ovariectomized mice in vivo (Fig. 5); and 3) HABP alone strongly inhibited bone formation parameters in vivo (Fig. 5), although it slightly increased OB differentiation from BM stromal cells in vitro (Fig. 1D), like nitrogen-containing BPs ^42^.

Our findings suggest that the administered dose of HABP, rather than having no or minimal antiresorptive effects, unexpectedly inhibited bone resorption, but the mechanism is different from that of conventional nitrogen-containing BPs. Nitrogen-containing BPs (N-BPs), like zoledronate, which impair OC resorptive functions and accelerate OC apoptosis, also increase OC numbers ^43^ because they induce an acute phase reaction and inhibit FPPS ^21,22^, resulting in disruption of cytoskeletal organization, loss of the ruffled border membrane, and altered vesicular trafficking ^23^. In contrast, the HABP inhibited OC formation, supported by our finding that it reduced OC numbers in vivo (Fig. 5B) without causing giant apoptotic OCs, as commonly occurs in animals (Fig. 4B) and humans treated with nitrogen-containing BPs ^43^. The effect of nitrogen-containing BPs to inhibit bone formation is attributed to their induction of farnesyl pyrophosphate accumulation in OBs due to the inhibition of FPPS activity ^44^ or is secondary to lower bone turnover. The HABP, in which the double side chains attached to the central carbon atom was replaced by one amido methylene chain, also inhibited bone formation, but we do not know if it inhibits FPPS activity. However, an amide function, rendering a nitrogen non-basic, further reduces any FPPS effect of a nitrogen in a bisphosphonate chain ^45,46^. The mild antiresorptive action of HABP appears to have enabled HCQ to better exert its anabolic function. This is different from the effects of nitrogen-containing BPs, such as alendronate and zoledronate, that strongly inhibit bone resorption and bone formation, thus disabling the effects of an anabolic agent ^15,17^. A high dose of HABP could potentially attenuate the anabolic effect of HCQ, as supported by our findings that the highest dose (20 mM/kg) of HABP-HCQ reduced OB surfaces in ovariectomized mice to the levels in vehicle-treated controls, despite restoring the lost bone along with high bone formation parameters. Thus, further studies will be required to determine the optimal combined dose of HABP and HCQ to increase bone formation and inhibit resorption in ovariectomized mice.

One of the major mechanisms for the dual antiresorptive and anabolic effects of HABP-HCQ in preventing and treating osteoporosis (Fig. 4&5) is to stabilize TRAF3, which limits OC formation and maintains OB differentiation ^28,30,31^. HABP-HCQ works similarly to HCQ to stabilize TRAF3 in both myeloid cells and MPCs, and thus it inhibits OC formation and stimulates OB differentiation, respectively, in vitro (Fig. 2A&B). However, the lowest dose of HCQ that inhibits OC formation in vitro is higher than can be administered in vivo without serious adverse effects, and thus the dose of HCQ we administered did not inhibit bone resorption parameters in vivo. The myeloid cell-specific TRAF3 cKO mice we used in this study have enhanced parameters of bone resorption, with normal bone formation (Fig. 4). HABP-HCQ, like zoledronic acid, inhibited bone resorption in these TRAF3 cKO mice because the HABP component of HABP-HCQ exerts most of the antiresorptive action of the combination, independent of TRAF3 in vivo. The HABP also prevented RANKL-induced TRAF3 degradation in OC precursors (Fig. 2A), and this mechanism may be related to its stimulation of OB differentiation in vitro (Fig. 2B).

Tissue fibrosis, characterized by the accumulation of extracellular matrix components, is a vital component of wound healing and tissue repair in response to injury, but it also occurs commonly in the elderly as a consequence of low-level of chronic inflammation of aging, particularly in fibrotic cardiac and respiratory diseases ^47^. BM fibrosis also occurs during aging ^48^, associated with osteoporosis and defective WNT1 signaling, characterized by increased reticulin deposition and altered granulopoiesis ^49^. However, the fibrosis is not correlated with the severity of osteoporosis ^49^. Inhibition of BM fibrosis could be one of the mechanisms whereby the HABP-HCQ prevents and reverses ovariectomy-induced bone loss, although there are no published data showing a relationship between BM fibrosis and osteoporosis during aging.

In summary, we found that a low dose of HCQ in combination with HABP has a dual anabolic and antiresorptive action, and thus prevented ovariectomy-induced bone loss in mice and restored the established bone loss caused by ovariectomy. It is possible that this combination could be used long-term and safely for the prevention and treatment of osteoporosis by avoiding the systemic side effects of a high dose of HCQ or a potent N-containing bisphosphonate.

## Experimental procedures

### Reagents:

Recombinant murine M-CSF and RANKL were purchased from R&D Systems (Minneapolis, MN). Hydroxychloroquine sulfate and zoledronic acid were purchased from Sigma. Recombinant human PTH (1–34) was purchased from GenScript. Abs against TRAF3 and actin were purchased from Santa Cruz.

### Synthesis of HABP:

An oven-dried round bottom flask was charged with a solution of tetraethyl (((4-hydroxybutanamido)methylene)bis(phosphonic acid)) **3** in anhydrous dichloromethane (0.4 g, 1.028 mmol in 2 mL). After cooling to 0°C, neat bromotrimethylsilane (0.8 mL) was added dropwise and the contents were carefully warmed to room temperature and stirred for 14 h. After concentrating under reduced pressure, the crude mixture was redissolved in methanol (2 mL) and concentrated. The procedure was repeated twice to yield 0.27 g (94.7 %) of BP (((4-hydroxybutanamido)methylene)bis(phosphonic acid))) as a white waxy paste.

^1^H-NMR (400 MHz, CD_3_OD) d 4.83 (t, *J* = 8 Hz, 1H), 3.47 (t, *J* = 5.6 Hz, 2H), 2.28 (t, *J* = 10 Hz, 2H), 1.85 – 1.67 (m, 2H). ^13^C-NMR (125 MHz, CD_3_OD) d 173.90, 61.44, 45.58 (t, *J* = 250 Hz), 31.23, 26.00. ^31^P-NMR (162 MHz, CD_3_OD) d 13.18. Thermo-MS (ESI) m/z (M + Na^+^) Calcd for C5H13NO8P2Na: 300.13 Found: 300.2.

### Animal surgery and drug administration:

All animal experimental protocols were approved by the University of Rochester Committee for Animal Resources, and all methods were carried out in accordance with the guidelines and regulations of the American Veterinary Medical Association (AVMA).

A multiple PTH injection resorption-inducing model was used to determine the effective dose of HCQ and HABP-HCQ to inhibit OC formation in vivo. Briefly, 2-mon-old female C57Bl6 mice were given vehicle, HCQ or HABP-HCQ daily for 6 d *via* intraperitoneal injection, and 2 μg recombinant PTH (1–34), 4 times per d, during the last 3 d (d 4 to 6) *via* SC injection over calvariae. The mice were euthanized on d 7 (last PTH injection was given 2 hr before death), and the calvarial and tibial bones were collected for processing and paraffin-embedded sectioning for TRAP and H&E staining to evaluate OC formation and BM fibrosis, respectively.

An ovariectomy (OVX) model was used to test if a low dose of HABP-HCQ is more effective than HCQ to prevent OVX-induced bone loss and if its effects depend on TRAF3 expression by OCs. 3-mon-old female mice with TRAF3 conditional knockout (cKO) in myeloid cells (TRAF3^f/f^LyM^cre^; TRAF3 cKO) and their littermate control (TRAF3^f/f^) mice ^31^ were randomly divided into 6 groups, 8–9 mice/group. One group had sham surgery and 5 groups of mice for each phenotype had OVX surgery, and were randomly assigned to vehicle, HCQ (20 mM/kg), HABP-HCQ (6 mM/kg), Zoledronic acid (Zol, 150 mg/kg) or PTH (80 ng/injection) treatment, starting on the 2^nd^ day after surgery. Vehicle, HCQ, or HABP-HCQ in a volume of 0.1 ml per 10 g body weight were given *via* daily IP injection, 5 doses/wk for 5 wk. Zol was given as a single IP injection weekly, while PTH was given as daily supra-calvarial SC injections, 5 doses/wk. To determine if a low dose of HABP-HCQ can treat established OVX-induced bone loss, 3-mon-old female C57Bl6 mice were ovariectomized and subsequently given different doses of HABP-HCQ, HCQ, HABP or PTH for 4 wk from the 5^th^ wk post-surgery when bone loss in the vertebrae of OVX mice was confirmed by micro-CT. The mice were given injections of calcein (5 mg/kg) on the 5^th^ and last day before sacrifice, following our standard protocol to assess bone formation parameters ^50,51^. After the mice were euthanized, the spinal bones were collected for micro-CT scanning, followed by histologic analysis of OBs and OCs in paraffin-embedded sections or dynamic bone formation parameters in plastic-embedded sections.

### Micro-CT evaluation:

spines including the lower part of thoracic to upper coccygeal vertebrae were fixed in 10% neutral phosphate-buffered formalin for 48 hr and were transferred to 70% ethanol at 4°C for storage. The spines were scanned using a vivaCT 40 instrument (Scanco Medical) at a voxel size of 7 μm, 50 kVp, 144 μA and 800 ms integration time. The machine was set at a threshold of 220 to distinguish bone from soft tissues. Cancellous bone in L1 vertebrae was assessed in 300 transverse slices to determine bone volume (BV/TV, %), trabecular thickness (Tb.Th, μm), trabecular number (Tb.N, #/mm), trabecular separation (Tb.Sp, μm), according to standard guidelines ^52^.

### Bone histomorphometric analysis:

After micro-CT scanning, L3-L5 vertebrae were decalcified for 3 wk using 10% EDTA at 4°C, processed, and embedded in paraffin. 3-μm-thick sections at the center of the vertebrae with fewest trabeculae, selected from a series of continuously cut sections to ensure the measurement of all the sections was comparable, were blindly quantified for histomorphometric parameters, including the structural trabecular bone parameters, BV/TV (%), Tb.Th (μm), Tb.N (#/mm), Tb.Sp (μm), and OB surfaces on H&E-stained sections, and OC parameters on TRAP-stained sections using an OsteoMeasure Image Analysis System (Osteometrics, Decatur, GA) ^50,51^ following the recommendations of the ASBMR Histomorphometry Nomenclature Committee ^53^. T12 to L2 vertebrae were processed as LR white plastic-embedded blocks, as we reported previously ^50,51^. 3 μm-thick plastic sections were cut using a carbide steel knife on a Shandon Microtome. Sections at the center of the vertebrae were collected to evaluate the dynamic parameters of bone formation using an OsteoMeasure Image Analysis System, as we reported previously ^50,51^, following the recommendations of the ASBMR Histomorphometry Nomenclature Committee ^53^.

### Osteoclastogenesis:

our culture procedure was modified from our previous reports ^50,51^. Briefly, we cut open both ends of each femur or tibia to expose the marrow cavity, flushed out BM with 10 ml of a-MEM containing 2% FBS by using a 21-gauge needle, and passed the cells through a 21-G needle 3 times to make single cell suspensions. The cells were incubated in NH_4_Cl solution for 15 min at room temperature to lyse red blood cells. 5×10^4^ cells were seeded in wells of 96-well plates with 5 ng/ml M-CSF for 2 d. Then RANKL (10 ng/ml) and different inhibitors were added to the cultures for an additional 2–3 d when mature OCs typically are observed under inverted microscopy. The cells were then fixed with 10% neutral, phosphate-buffered formalin for 10 min and stained for TRAP activity. TRAP^+^ cells with 3 or more nuclei were considered as mature OCs.

### In vitro osteoblast differentiation assay:

1×10^6^ BM cells from WT mice were seeded in 12-well-plates with a-MEM containing 15% FBS for 5 d followed by induction of OB differentiation with 25 μg/ml ascorbic acid and 5 mM β-glycerophosphate ^50,51^. The cells were fixed after 7 d with 10% neutral phosphate-buffered formalin followed by ALP or after 14d followed by Von Kossa staining to measure the area (Ar.) of ALP^+^ cells and mineralized nodules, respectively. Similarly, bone-derived mesenchymal progenitor cells (BdMPCs) from WT mice ^50,51^ were used to test the effect of compounds on OB differentiation. Briefly, BdMPCs were seeded in 12-well plates, 1×10^4^/well. From the second day, the cells were induced for OB differentiation, as above, in the presence of different compounds for 7 d. ALP^+^ OB differentiation was evaluated using ALP staining.

### Western Blot analysis:

macrophages induced by M-CSF from C57Bl6 mouse BM and C3H10T1/2 mouse MSCs, treated with different compounds, were lysed with M-Per mammalian protein extraction reagent (Thermo Scientific) containing a protease inhibitor cocktail (Sigma). Lysates (10–20 μg) were loaded in 10% SDS-PAGE gels and transferred onto polyvinylidene difluoride membranes. Following blocking in 5% milk, membranes were incubated overnight at 4°C with anti-mouse TRAF3 or b-actin Ab. After washing, the membranes were incubated with horseradish peroxidase-linked secondary Ab (Bio-Rad). The membranes were exposed to ECL substrate and signals were analyzed using a Bio-Rad imaging system.

### Statistics.

All data had a normality test. Data are given as the mean ± S.D. when they were distributed normally. Median and interquartile ranges were used instead when data distributions were skewed. Comparisons between 2 groups were analyzed using Student’s two-tailed unpaired *t*-test and those among 3 or more groups using one-way analysis of variance followed by Dunnett’s post-hoc multiple comparisons when data were distributed normally. In contrast, log transformed data were used to do statistical analyses when data distributions were skewed. *p* values <0.05 were considered statistically significant. Each in vitro experiment was repeated 3 times with similar results.

## Abbreviations and nomenclature

OVX: Ovariectomy
OC: Osteoclast
OB: Osteoblast
BPs: Bisphosphonates
BdMPCs: Bone-derived mesenchymal progenitor cells
BMD: Bone mineral density
BV/TV: Bone volume/tissue volume
Tb.Th: Trabecular thickness
Tb.N: Trabecular number
Tb.Sp: Trabecular separation
ALP: Alkaline phosphatase
TRAP: Tartrate-resistant acid phosphatase
BM: Bone marrow
BFR: Bone formation rate
MAR: Mineral apposition rate
RANKL: Receptor activator of nuclear factor kappa-Β ligand

## References

[1] Looker A. C., Melton L. J. 3rd, Harris T. B., Borrud L. G. & Shepherd J. A. Prevalence and trends in low femur bone density among older US adults: NHANES 2005–2006 compared with NHANES III. J Bone Miner Res 25, 64–71 (2010). 10.1359/jbmr.090706 19580459 PMC3312738

[2] Baghdadi S. Mortality following proximal femoral fractures in elderly patients: a large retrospective cohort study of incidence and risk factors. BMC Musculoskelet Disord 24, 693 (2023). 10.1186/s12891-023-06825-9 37649030 PMC10466793

[3] Mashiba T. Suppressed bone turnover by bisphosphonates increases microdamage accumulation and reduces some biomechanical properties in dog rib. J Bone Miner Res 15, 613–620 (2000). 10.1359/jbmr.2000.15.4.613 10780852

[4] Eastell R. Effects of denosumab on bone turnover markers in postmenopausal osteoporosis. J Bone Miner Res 26, 530–537 (2011). 10.1002/jbmr.251 20839290

[5] Wasnich R. D. & Miller P. D. Antifracture efficacy of agents are related to changes in bone density. J Clin Endocrinol Metab 85, 231–236 (2000). 10.1210/jcem.85.1.6267 10634392

[6] Khan A. A. Diagnosis and management of osteonecrosis of the jaw: a systematic review and international consensus. J Bone Miner Res 30, 3–23 (2015). 10.1002/jbmr.2405 25414052

[7] Cummings S. R. Vertebral Fractures After Discontinuation of Denosumab: A Post Hoc Analysis of the Randomized Placebo-Controlled FREEDOM Trial and Its Extension. J Bone Miner Res 33, 190–198 (2018). 10.1002/jbmr.3337 29105841

[8] Anastasilakis A. D. Denosumab Discontinuation and the Rebound Phenomenon: A Narrative Review. J Clin Med 10 (2021). 10.3390/jcm10010152 PMC7796169 33406802

[9] Ponnapakkam T., Katikaneni R., Sakon J., Stratford R. & Gensure R. C. Treating osteoporosis by targeting parathyroid hormone to bone. Drug Discov Today 19, 204–208 (2014). 10.1016/j.drudis.2013.07.015 23932952 PMC3979969

[10] Cipriani C., Irani D. & Bilezikian J. P. Safety of osteoanabolic therapy: a decade of experience. J Bone Miner Res 27, 2419–2428 (2012). 10.1002/jbmr.1800 23165426

[11] Cosman F. Romosozumab Treatment in Postmenopausal Women with Osteoporosis. N Engl J Med 375, 1532–1543 (2016). 10.1056/NEJMoa1607948 27641143

[12] Sleeman A. & Clements J. N. Abaloparatide: A new pharmacological option for osteoporosis. Am J Health Syst Pharm 76, 130–135 (2019). 10.1093/ajhp/zxy022 30689744

[13] Solling A. S. K., Harslof T. & Langdahl B. Current Status of Bone-Forming Therapies for the Management of Osteoporosis. Drugs Aging 36, 625–638 (2019). 10.1007/s40266-019-00675-8 31066015

[14] Leder B. Z., Tsai J. N., Jiang L. A. & Lee H. Importance of prompt therapy in postmenopausal women discontinuing teriparatide or denosumab: The Denosumab and Teriparatide Follow-up study (DATA-Follow-up). Bone 98, 54–58 (2017). 10.1016/j.bone.2017.03.006 28286299

[15] Black D. M. The effects of parathyroid hormone and alendronate alone or in combination in postmenopausal osteoporosis. N Engl J Med 349, 1207–1215 (2003). 10.1056/NEJMoa031975 14500804

[16] Finkelstein J. S. The effects of parathyroid hormone, alendronate, or both in men with osteoporosis. N Engl J Med 349, 1216–1226 (2003). 10.1056/NEJMoa035725 14500805

[17] Cosman F., Nieves J. W. & Dempster D. W. Treatment Sequence Matters: Anabolic and Therapy for Osteoporosis. J Bone Miner Res 32, 198–202 (2017). 10.1002/jbmr.3051 27925287

[18] Chavassieux P. Bone-Forming and Effects of Romosozumab in Postmenopausal Women With Osteoporosis: Bone Histomorphometry and Microcomputed Tomography Analysis After 2 and 12 Months of Treatment. J Bone Miner Res 34, 1597–1608 (2019). 10.1002/jbmr.3735 31233639 PMC7027577

[19] Shoback D. Pharmacological Management of Osteoporosis in Postmenopausal Women: An Endocrine Society Guideline Update. J Clin Endocrinol Metab 105 (2020). 10.1210/clinem/dgaa048 32068863

[20] Rogers M. J., Monkkonen J. & Munoz M. A. Molecular mechanisms of action of bisphosphonates and new insights into their effects outside the skeleton. Bone 139, 115493 (2020). 10.1016/j.bone.2020.115493 32569873

[21] van Beek E., Pieterman E., Cohen L., Lowik C. & Papapoulos S. Farnesyl pyrophosphate synthase is the molecular target of nitrogen-containing bisphosphonates. Biochem Biophys Res Commun 264, 108–111 (1999). 10.1006/bbrc.1999.1499 10527849

[22] Tsoumpra M. K. The inhibition of human farnesyl pyrophosphate synthase by nitrogen-containing bisphosphonates. Elucidating the role of active site threonine 201 and tyrosine 204 residues using enzyme mutants. Bone 81, 478–486 (2015). 10.1016/j.bone.2015.08.020 26318908 PMC4652608

[23] Brown J. P. Long-Term Treatment of Postmenopausal Osteoporosis. Endocrinol Metab (Seoul) 36, 544–552 (2021). 10.3803/EnM.2021.301 34154042 PMC8258325

[24] Dunford J. E. Structure-activity relationships for inhibition of farnesyl diphosphate synthase in vitro and inhibition of bone resorption in vivo by nitrogen-containing bisphosphonates. J Pharmacol Exp Ther 296, 235–242 (2001). 11160603

[25] Xing L. Targeting anti-cancer agents to bone using bisphosphonates. Bone 138, 115492 (2020). 10.1016/j.bone.2020.115492 32585321 PMC8485333

[26] Sun S. Bisphosphonates for delivering drugs to bone. Br J Pharmacol 178, 2008–2025 (2021). 10.1111/bph.15251 32876338 PMC8515266

[27] Yao Z. RANKL cytokine enhances TNF-induced osteoclastogenesis independently of TNF receptor associated factor (TRAF) 6 by degrading TRAF3 in osteoclast precursors. J Biol Chem 292, 10169–10179 (2017). 10.1074/jbc.M116.771816 28438834 PMC5473222

[28] Yao Z., Xing L. & Boyce B. F. NF-kappaB p100 limits TNF-induced bone resorption in mice by a TRAF3-dependent mechanism. J Clin Invest 119, 3024–3034 (2009). 10.1172/JCI38716 19770515 PMC2752069

[29] Ni C. Z. Molecular basis for CD40 signaling mediated by TRAF3. Proc Natl Acad Sci U S A 97, 10395–10399 (2000). 10.1073/pnas.97.19.10395 10984535 PMC27035

[30] Li J. TGFbeta-induced degradation of TRAF3 in mesenchymal progenitor cells causes age-related osteoporosis. Nat Commun 10, 2795 (2019). 10.1038/s41467-019-10677-0 31243287 PMC6595054

[31] Xiu Y. Chloroquine reduces osteoclastogenesis in murine osteoporosis by preventing TRAF3 degradation. J Clin Invest 124, 297–310 (2014). 10.1172/JCI66947 24316970 PMC3871219

[32] Motten A. G. Photophysical studies on antimalarial drugs. Photochem Photobiol 69, 282–287 (1999). 10089818 10.1562/0031-8655(1999)069<0282:psoad>2.3.co;2

[33] Costedoat-Chalumeau N. A Critical Review of the Effects of Hydroxychloroquine and Chloroquine on the Eye. Clin Rev Allergy Immunol (2015). 10.1007/s12016-015-8469-8 25672591

[34] Silva B. C. & Bilezikian J. P. Parathyroid hormone: anabolic and catabolic actions on the skeleton. Curr Opin Pharmacol 22, 41–50 (2015). 10.1016/j.coph.2015.03.005 25854704 PMC5407089

[35] Rosenbaum J. T. American College of Rheumatology, American Academy of Dermatology, Rheumatologic Dermatology Society, and American Academy of Ophthalmology 2020 Joint Statement on Hydroxychloroquine Use With Respect to Retinal Toxicity. Arthritis Rheumatol 73, 908–911 (2021). 10.1002/art.41683 33559327

[36] Nair A. B. & Jacob S. A simple practice guide for dose conversion between animals and human. J Basic Clin Pharm 7, 27–31 (2016). 10.4103/0976-0105.177703 27057123 PMC4804402

[37] Rogers M. J., Crockett J. C., Coxon F. P. & Monkkonen J. Biochemical and molecular mechanisms of action of bisphosphonates. Bone 49, 34–41 (2011). 10.1016/j.bone.2010.11.008 21111853

[38] Jobke B., Milovanovic P., Amling M. & Busse B. Bisphosphonate-osteoclasts: changes in osteoclast morphology and function induced by nitrogen-containing bisphosphonate treatment in osteoporosis patients. Bone 59, 37–43 (2014). 10.1016/j.bone.2013.10.024 24211427

[39] Coxon F. P. Protein geranylgeranylation is required for osteoclast formation, function, and survival: inhibition by bisphosphonates and GGTI-298. J Bone Miner Res 15, 1467–1476 (2000). 10.1359/jbmr.2000.15.8.1467 10934645

[40] Coxon F. P., Thompson K. & Rogers M. J. Recent advances in understanding the mechanism of action of bisphosphonates. Curr Opin Pharmacol 6, 307–312 (2006). 10.1016/j.coph.2006.03.005 16650801

[41] Halasy-Nagy J. M., Rodan G. A. & Reszka A. A. Inhibition of bone resorption by alendronate and risedronate does not require osteoclast apoptosis. Bone 29, 553–559 (2001). 10.1016/s8756-3282(01)00615-9 11728926

[42] Maruotti N., Corrado A., Neve A. & Cantatore F. P. Bisphosphonates: effects on osteoblast. Eur J Clin Pharmacol 68, 1013–1018 (2012). 10.1007/s00228-012-1216-7 22318756

[43] Weinstein R. S., Roberson P. K. & Manolagas S. C. Giant osteoclast formation and long-term oral bisphosphonate therapy. N Engl J Med 360, 53–62 (2009). 10.1056/NEJMoa0802633 19118304 PMC2866022

[44] Weivoda M. M. & Hohl R. J. Effects of farnesyl pyrophosphate accumulation on calvarial osteoblast differentiation. Endocrinology 152, 3113–3122 (2011). 10.1210/en.2011-0016 21586555

[45] Ebetino F. H. The relationship between the chemistry and biological activity of the bisphosphonates. Bone 49, 20–33 (2011). 10.1016/j.bone.2011.03.774 21497677

[46] Ebetino F. H. Bisphosphonates: The role of chemistry in understanding their biological actions and structure-activity relationships, and new directions for their therapeutic use. Bone 156, 116289 (2022). 10.1016/j.bone.2021.116289 34896359 PMC11023620

[47] Murtha L. A. The Role of Pathological Aging in Cardiac and Pulmonary Fibrosis. Aging Dis 10, 419–428 (2019). 10.14336/AD.2018.0601 31011486 PMC6457057

[48] Wang Y., Wehling-Henricks M., Samengo G. & Tidball J. G. Increases of M2a macrophages and fibrosis in aging muscle are influenced by bone marrow aging and negatively regulated by muscle-derived nitric oxide. Aging Cell 14, 678–688 (2015). 10.1111/acel.12350 26009878 PMC4531081

[49] Makitie R. E. Defective WNT signaling associates with bone marrow fibrosis-a cross-sectional cohort study in a family with WNT1 osteoporosis. Osteoporos Int 29, 479–487 (2018). 10.1007/s00198-017-4309-4 29147753

[50] Shen G., Liu X., Lei W., Duan R. & Yao Z. Plumbagin is a NF-kappaB-inducing kinase inhibitor with dual anabolic and effects that prevents menopausal-related osteoporosis in mice. J Biol Chem 298, 101767 (2022). 10.1016/j.jbc.2022.101767 35235833 PMC8958545

[51] Yi X. TNF-Polarized Macrophages Produce Insulin-like 6 Peptide to Stimulate Bone Formation in Rheumatoid Arthritis in Mice. J Bone Miner Res (2021). 10.1002/jbmr.4447 PMC8688308 34585777

[52] Bouxsein M. L. Guidelines for assessment of bone microstructure in rodents using micro-computed tomography. J Bone Miner Res 25, 1468–1486 (2010). 10.1002/jbmr.141 20533309

[53] Dempster D. W. Standardized nomenclature, symbols, and units for bone histomorphometry: a 2012 update of the report of the ASBMR Histomorphometry Nomenclature Committee. J Bone Miner Res 28, 2–17 (2013). 10.1002/jbmr.1805 23197339 PMC3672237

